# 3D nanomechanical mapping of subcellular and sub-nuclear structures of living cells by multi-harmonic AFM with long-tip microcantilevers

**DOI:** 10.1038/s41598-021-04443-w

**Published:** 2022-01-11

**Authors:** Yuri M. Efremov, Daniel M. Suter, Peter S. Timashev, Arvind Raman

**Affiliations:** 1 School of Mechanical Engineering, Purdue University, West Lafayette, IN USA; 2 Birck Nanotechnology Center, Purdue University, West Lafayette, IN USA; 3 Institute for Regenerative Medicine, Sechenov University, Moscow, Russia; 4 World-Class Research Center “Digital Biodesign and Personalized Healthcare, Moscow, Russia; 5 Department of Biological Sciences, Purdue University, West Lafayette, IN USA; 6 Bindley Bioscience Center, Purdue University, West Lafayette, IN USA; 7 Purdue Institute for Integrative Neuroscience, West Lafayette, IN USA; 8 Chemistry Department, Lomonosov Moscow State University, Moscow, Russia

**Keywords:** Biophysics, Nanoscale biophysics, Atomic force microscopy, Cellular imaging

## Abstract

Recent developments such as multi-harmonic Atomic Force Microscopy (AFM) techniques have enabled fast, quantitative mapping of nanomechanical properties of living cells. Due to their high spatiotemporal resolution, these methods provide new insights into changes of mechanical properties of subcellular structures due to disease or drug response. Here, we propose three new improvements to significantly improve the resolution, identification, and mechanical property quantification of sub-cellular and sub-nuclear structures using multi-harmonic AFM on living cells. First, microcantilever tips are streamlined using long-carbon tips to minimize long-range hydrodynamic interactions with the cell surface, to enhance the spatial resolution of nanomechanical maps and minimize hydrodynamic artifacts. Second, simultaneous Spinning Disk Confocal Microscopy (SDC) with live-cell fluorescent markers enables the unambiguous correlation between observed heterogeneities in nanomechanical maps with subcellular structures. Third, computational approaches are then used to estimate the mechanical properties of sub-nuclear structures. Results are demonstrated on living NIH 3T3 fibroblasts and breast cancer MDA-MB-231 cells, where properties of nucleoli, a deep intracellular structure, were assessed. The integrated approach opens the door to study the mechanobiology of sub-cellular structures during disease or drug response.

## Introduction

Changes in the mechanical properties of whole living cells are known to be closely related to a large variety of physiological and pathological processes^1–6^. However, much less is known about the corresponding changes in the mechanical properties of individual subcellular components including organelles. Yet, the changes in the properties of these components might precede the change in global cellular properties and serve as valuable prognostic markers. Molecular and structural alterations of subcellular components like the cytoskeleton^7,8^, nucleus, and nucleolus are known to be a marker of diseases^9,10^. One way to characterize subcellular organelles involves their isolation and separation from other components^11,12^. This, however, leads to complicated preparation steps and raises questions about the preservation of native properties of the isolated components.

Several new techniques have recently emerged to characterize the mechanical properties of the subcellular components directly inside living cells. One such method, optics-based noninvasive Brillouin microscopy, has been used to quantify the mechanical properties of plant extracellular matrices^13^ and mammalian cells^14^ at the submicrometer scale. Other methods involve particle tracking inside the cell to measure the local viscoelastic properties of the cytoplasm^15^.

AFM has been successfully used in recent years to characterize the mechanical properties of cells^16–18^. One of the limitations of this technique is that the cantilever interacts with the surface of the cell, and that the obtained data describe the topmost sublayers only. Thus, an information about subcellular structures that are localized deeper below the cell surface including cytoskeletal elements, nucleus, mitochondria, lysosomes, and Golgi apparatus generally remains unavailable.

To address this challenge, many efforts have been made recently to image sub-surface features through tip-generated stress or electric fields in AFM^19–21^, some of which might be applied to the living cells^22–25^. Nanomechanical holography, a method involving ultrasonic waves that are detected by an AFM probe in contact with the cell surface, has been applied to image the inner structures of cells and to detect the presence of nanoparticles inside the cells of exposed animals^24^. However, difficulties in interpreting the images in terms of the mechanical properties of the subsurface structures limit the broader application of this technique. Force-spectroscopy-based methods were also suggested, where each force curve is divided by sections based on the indentation depth. Each section is then processed by using a linear elastic^23^ or viscoelastic model^22^ to analyze in-depth properties of the sample. These techniques, however, have limited time-resolution due to the acquisition of many force curves. Subcellular features have often been observed with Peak Force Tapping^26,27^ or dAFM^28,29^ methods. These prior techniques are generally limited either in spatial, or temporal resolution while imaging live cells or are unable to quantify the properties of the sub-cellular object being imaged.

We have recently developed a multi-harmonic dynamic AFM (dAFM) method to map quantitatively the nanomechanical properties of soft biological samples in a liquid environment at kHz frequencies^29–32^, which is compatible with commercial AFM systems equipped with a direct-excitation setup. Here, we demonstrate that with some key additional advances this method provides unprecedented observations of the deep intracellular structures (nucleoli) and suggest a way to quantitatively extract the mechanical properties of these subcellular structures. Three new approaches were combined here: (1) microcantilever with long-carbon tips to minimize hydrodynamic artifacts and improve the spatial resolution; (2) simultaneous imaging with spinning disk confocal (SDC) microscopy to confirm the identity of intracellular structures, their size and location in the living cell; (3) computational approaches to estimate the mechanical properties of subcellular structures.

## Results and discussion

### Cantilevers with long tips to improve nanomechanical contrast

Quantitative multi-harmonic imaging on living cells first and foremost requires microcantilevers that are directly excited, i.e. the excitation force is applied only to the microcantilever^33–36^. This includes magnetic, Lorenz force, piezoelectric, and photothermal excitation methods. The use of directly excited AFM microcantilevers or multi-harmonic AFM of living cells has been described previously^30,31^. Here, we will focus on the implementation that uses deflection feedback (0th harmonic feedback) on living cells. This approach results in an order of magnitude improvement in scanning speed when compared to the traditional feedback on the first harmonic amplitude^28,29^.

The main principles of application of dAFM (AM-AFM) technique with deflection feedback on very soft samples such as living cells (modulus < 1 MPa) were considered previously^28,30,31^. At the imaging set point for deflection (*A*_*0*_), the tip is not intermittently tapping the cell but rather permanently interacts with it over the entire oscillation cycle. Thus, higher harmonics (2nd and beyond) of microcantilever vibration are usually not observed while utilizing resonant microcantilevers for imaging mammalian cells; however higher harmonics are observed for stiffer bacterial cells and viruses^31,32^ where the tip intermittently taps the sample. The multi-harmonic observables (amplitudes *A*_*0*_, *A*_1_, and phase *φ*_*1*_) acquired on a living cell can be related to the local viscoelastic properties. The tip oscillation amplitude is small compared to the average indentation, which allows the use of contact mechanics models such as Hertz’s^37^, Sneddon’s^38^, or models with bottom effect correction^39,40^. From these mechanical models, the local storage *E*_*storage*_ and loss *E*_*loss*_ Young’s moduli can be obtained. However, as shown in^30,31^, it is important to consider the hydrodynamic coupling between the cantilever and the sample for correct data processing. The developed theory is described in the Supplementary Material.

When any dAFM technique is used in liquid, the squeeze-film hydrodynamic effect between the oscillating cantilever, the surrounding liquid, and a sample surface reduces the quality factor of the cantilever^30,41–43^ as well as its natural frequency. The oscillation amplitude decreases while the phase lag increases as the resonant cantilever approaches the surface. The extent of this effect depends on the oscillation frequency (through the unsteady Reynold’s number) and the gap (through the squeeze number)^41,43^. As a rule of thumb for AFM cantilevers oscillating near hard surfaces, the hydrodynamic effect generally starts to be noticeable when the gap becomes comparable to the cantilever half-width^41,43^, which is usually several 10’s of micrometers. Less studied but equally important is that the squeeze film effect also depends on the viscoelastic properties of the surface^44^ approached by the AFM microcantilever in liquid. In turn, the viscoelastic cell properties also depend on the height of the cell surface from the substrate, which changes from the nuclear region to the periphery of the cell^40^. This leads to two distinct artifacts that challenge quantitative dAFM methods on living cells:

- The tip height for soft AFM cantilevers usually used for imaging living cells are small (of the order of 3–5 µm). Thus, when the AFM tip contacts a living cell in liquid, the main AFM cantilever body is well within the range to experience significant squeeze film damping. As a result, the harmonic observables *A*_*near*_ and *φ*_*near*_ right before the tip-surface contact can be substantially different from the values *A*_*far*_ and *φ*_*far*_ several hundred microns above the surface where cantilever tuning is usually performed. this difference cannot be attributed to local tip-sample interaction properties but rather to the long-range hydrodynamic effect.
- The hydrodynamic coupling to the surface and the resulting resonant cantilever’s *A*_*near*_ and *φ*_*near*_ values are different on cells as compared to when the cantilever is located at the same height above a rigid surface^30^. The coupling differs even when the cantilever is scanning at the same height above different regions of the same cell (nucleus region versus periphery).

Thus, the hydrodynamic squeeze film effect acts as a long-range interaction between the cantilever body and the surface which significantly reduces the method’s resolution. Moreover, the dependence of *A*_*near*_ and *φ*_*near*_ values on the effective viscoelastic properties of the surface over which the cantilever is located can cause significant local mechanical property/topography-associated artifacts in the observables even before the tip contacts the surface.

**Figure 1:**
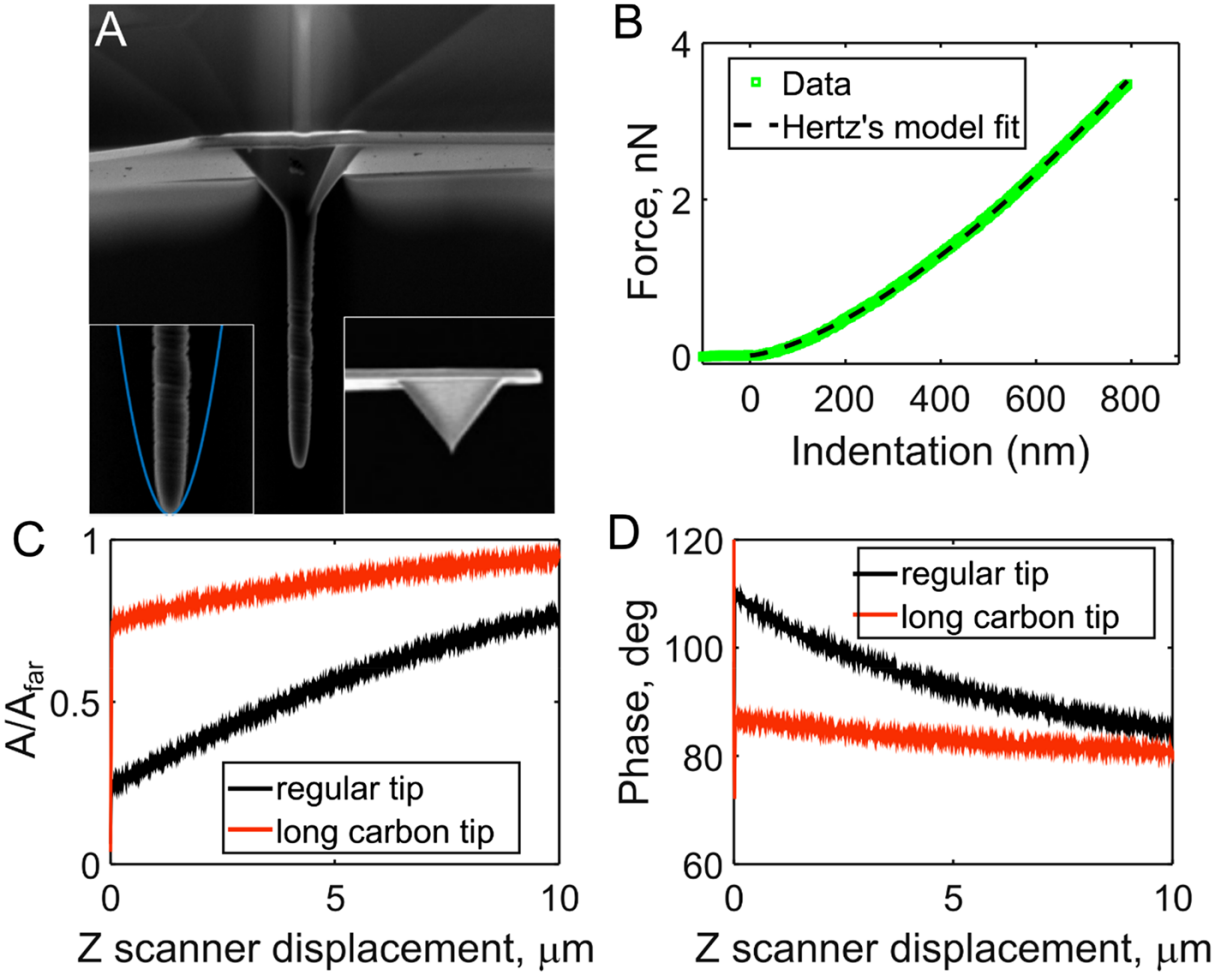
Cantilevers with long carbon tips. (**A**) SEM image (left inset—a fit with parabola to estimate the tip radius; right inset—a regular tip of the same cantilever type). (**B**) Force vs. Indentation curve obtained on the hydrogel fit well with the Hertz’s contact model (paraboloid). (**C**) Normalized amplitude vs. distance from the surface (glass) in the liquid (PBS). (**D**) Phase vs. distance from the surface (glass) in liquid (PBS).

Since most of this hydrodynamic artifact arises from the coupling of the cantilever beam and the surface, the tip height serves as an offset, which can control a magnitude of the effect. To reduce the squeeze-film hydrodynamic effect and boost the resolution of sub-cellular imaging, we grew 10–15 µm long high aspect ratio carbon tips on the 3–5 µm tip of BL-TR400PB cantilevers (Fig. 1A), thus increasing the tip height by 300–500%. The radius of carbon tips was 50–300 nm (150 nm typical), which is large enough to reduce the possibility of cell membrane penetration and damage. No cell damage was observed from comparing confocal imaging before and after the scanning (Fig. S1).

**Figure 2:**
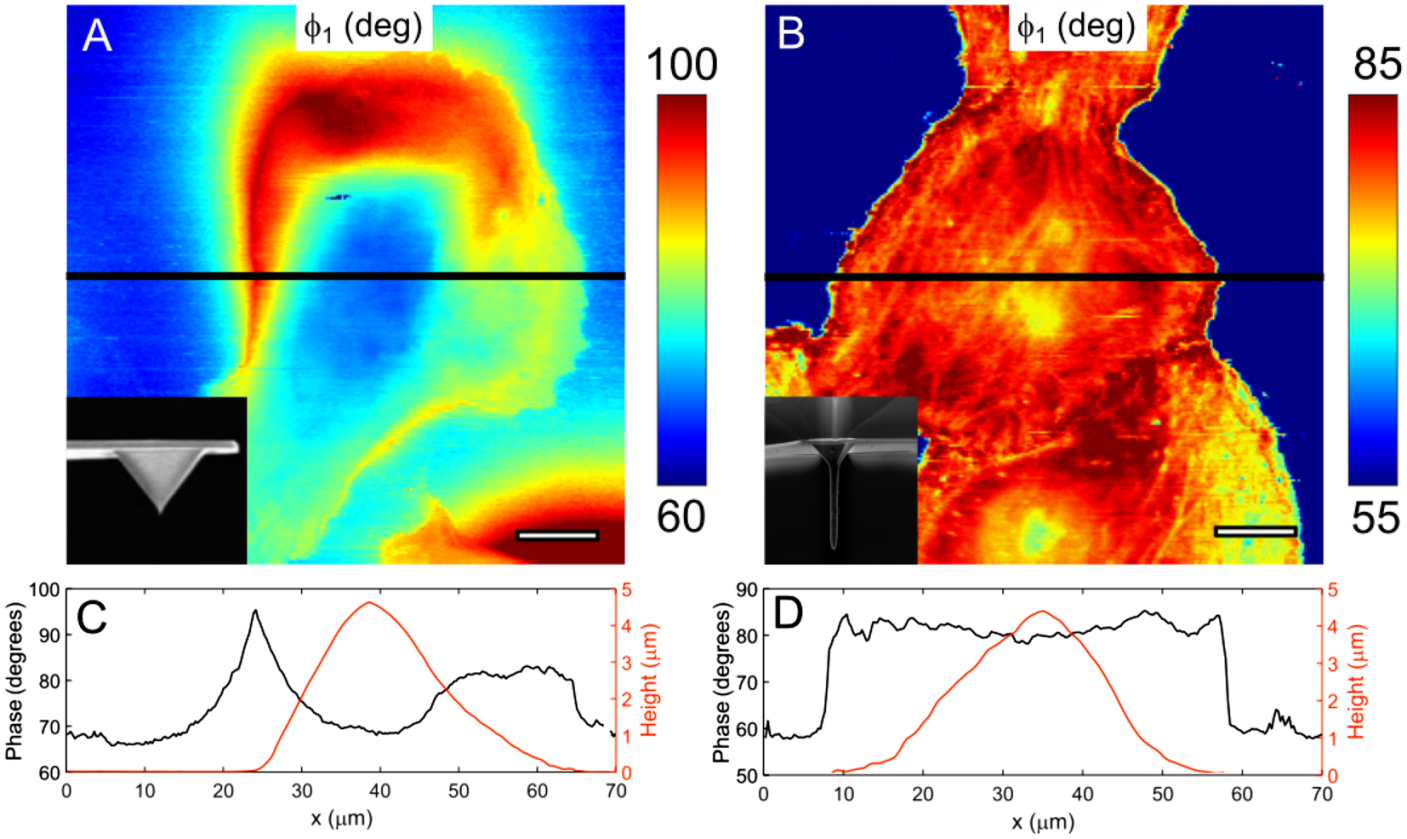
Cantilevers with long tip reduce topography convolution observed in phase images. (**A**). Phase image of a tall (~ 5 µm) MDA-MB-231 cell. Phase increase beyond the cell is seen in the top part of the image caused by the hydrodynamic interaction between the cell surface and the cantilever beam behind the tip. (**B**) This effect is not presented when using the long tips; the contrast is higher, cytoskeleton fibers and nucleoli could be distinguished. Insets: SEM image of the tip. (**C**) Cross-section of the phase signal along the black line indicated in (**A**) showing the effect of the phase signal affected by the topography for the standard tip. The phase value is lower over the highest part of the cell (lower hydrodynamic drag). (**D**) Cross-section of the phase signal over the marked line in (**B**), long tip. The phase does not noticeably correlate with the topography.

As can be seen in Fig. 1C,D for these long-tipped AFM microcantilevers the squeeze film induced *φ*_*far*_*—φ*_*near*_ (10 vs. 35 degrees for the standard microcantilevers) and *A*_*near*_/*A*_*far*_ ratio (0.74 vs. 0.24 for the standard cantilever) are significantly reduced in PBS on a glass surface and also on hydrogel and cell (Fig. S2). The reduction in hydrodynamic effect also led to weaker topography dependence of *φ*_*near*_ (Fig. 2C,D) and *A*_*near*_. Specifically, when standard tips were used, the phase signal *φ*_*1*_*,* which is affected by *φ*_*near*_, showed a significant negative correlation with the cell height (p < 0.001, Pearson correlation test for the part of the curves over the cell on Fig. 2C). No such correlation (p = 0.5) was found for the long-tipped AFM microcantilevers (Fig. 2D), which confirms the artificial origin of the correlation observed for standard tips. Longer tip AFM cantilever also ensure that the amplitude and phase variables change during a scan solely due to local tip-sample interactions. This leads to higher spatial contrast in dAFM observables as well as more reliable nano-mechanical maps. In particular, the small actin cytoskeleton elements could be distinguished much better in the phase signal (Fig. 2), which consequently led to the better resolved nanomechanical maps (Figs. 2B,D, 4A). Moreover, the long tips with high aspect-ratio are generally helpful to observe tall objects like cells to avoid possible interactions between the tip sides or cantilever beam and the cell surface. Not only do cantilever body-cell interactions lead to image distortions, but small cantilever-cell gaps cause a change in the hydrodynamic loading. The latter fact is observed as an increase in the phase beyond the tall cell (with respect to the cantilever beam axis, Fig. 2A).

The modified cantilevers were characterized by laser Doppler vibrometry to estimate the spring constant from thermal vibrations in air using the equipartition theorem. The tip shape was characterized using SEM images (Fig. 1A), the tip radius was estimated by a fit with a parabolic function (Fig. 1A, inset). The indentation of polyacrylamide gels with known mechanical properties was performed to confirm the contact geometry. Generally, the obtained force curves fit well (Adjusted R squared > 0.95) with the contact model for the paraboloid (Hertz model) (Fig. 1B). The Young’s modulus of the polyacrylamide gel measured with the modified cantilevers was within 5% of the modulus measured with colloidal probes (5 µm diameter).

The idea of using long tips is not novel by itself and such cantilevers were used before, for example, to improve the output of the contact resonance^45^, Force Mapping^46^, PeakForce Tapping^47,48^, and high-speed AFM^49^. Extra-long cantilever tips (~ 60 µm or 100–300 µm) have been used to keep the cantilever beam from submerging into the liquid during the scanning, thus removing most of the hydrodynamic damping and preserving high Q-factor (100) and operational frequencies (hundreds of kHz)^50,51^. However, they require low (50–100 μm) and controllable thickness of the liquid layer above the sample surface. In the method described here, the cantilevers are fully immersed into the liquid and thus have low Q-factor and operating frequencies. Our goal with this design is to increase the resolution of sub-cellular imaging using multi-harmonic AFM. This approach simplifies the conversion of the observables to local mechanical properties and facilitates comparison with the other AFM methods where low frequencies are used as well. The viscoelastic behavior of living cells is known to vary widely depending on the frequency range^52^. Other methods, like Brillouin microscopy, operate at much higher frequencies (GHz range) at which origin of the contrast and relevance to the cell physiological behavior is less studied. Also, the spatial resolution of Brillouin images is currently not high enough to see the individual actin stress fibers^14^.

### Observation of subcellular and subnuclear structures with improved contrast

**Figure 3:**
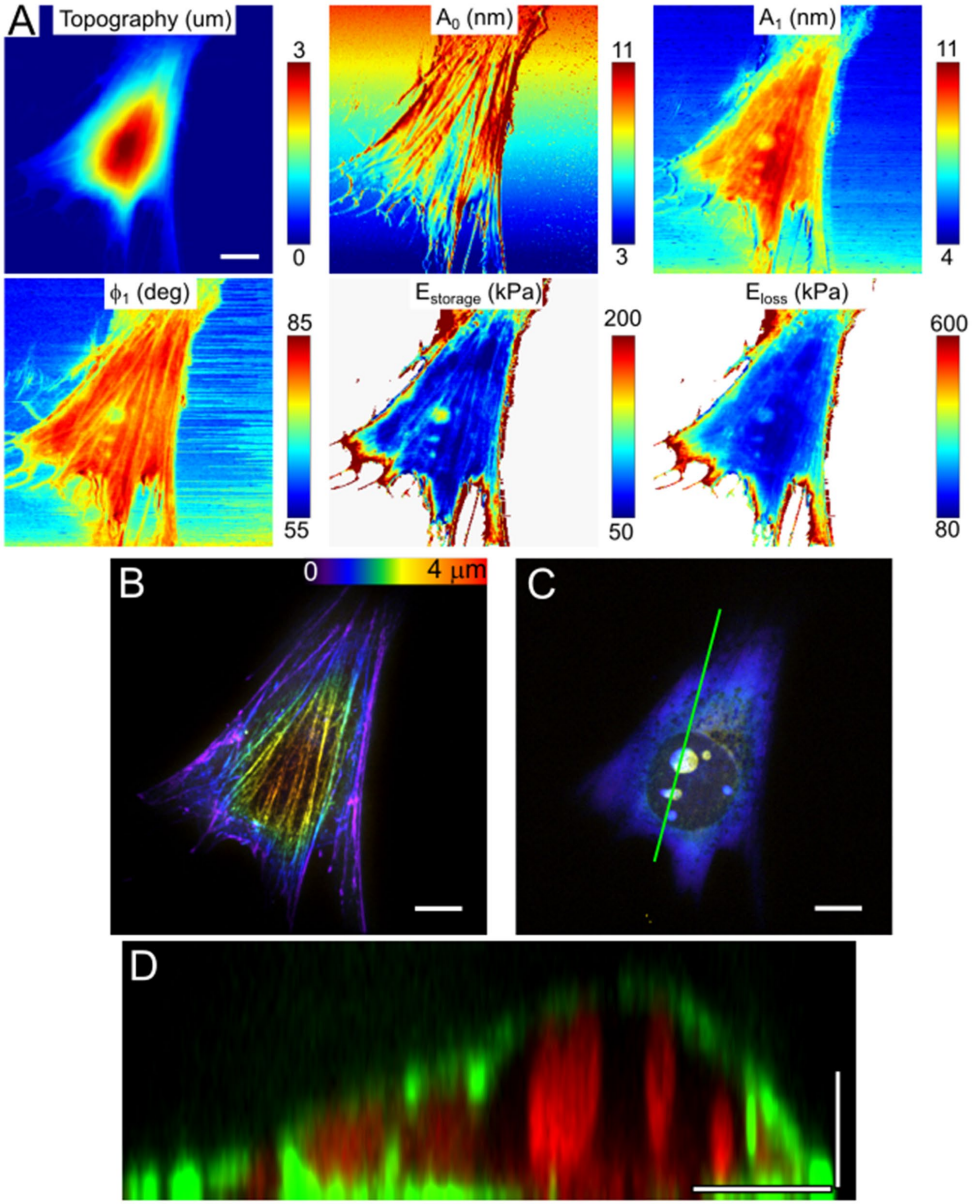
The NIH 3T3 fibroblast, AFM observables and nanomechanical maps. (**A**) and SDCM color-coded height images (**B**,**C**). Both perinuclear actin cap fibers and nucleoli could be detected in the amplitude and phase images, and in the E_storage_ map. Only the actin fibers, but not nucleoli, are presented in topography and deflection images. (**B**,**C**) Corresponding images of the F-actin cytoskeleton (SiR-actin staining, **B**) and nucleoli (SYTO85 staining, **C**), color-coded Z-stacks. (**D**) Vertical cross-section along the marked line shows the size and depth of the nucleoli (red) and cell shape (F-actin, green). Scale bars are 10 μm in the horizontal direction and 2 μm in the vertical direction.

**Figure 4:**
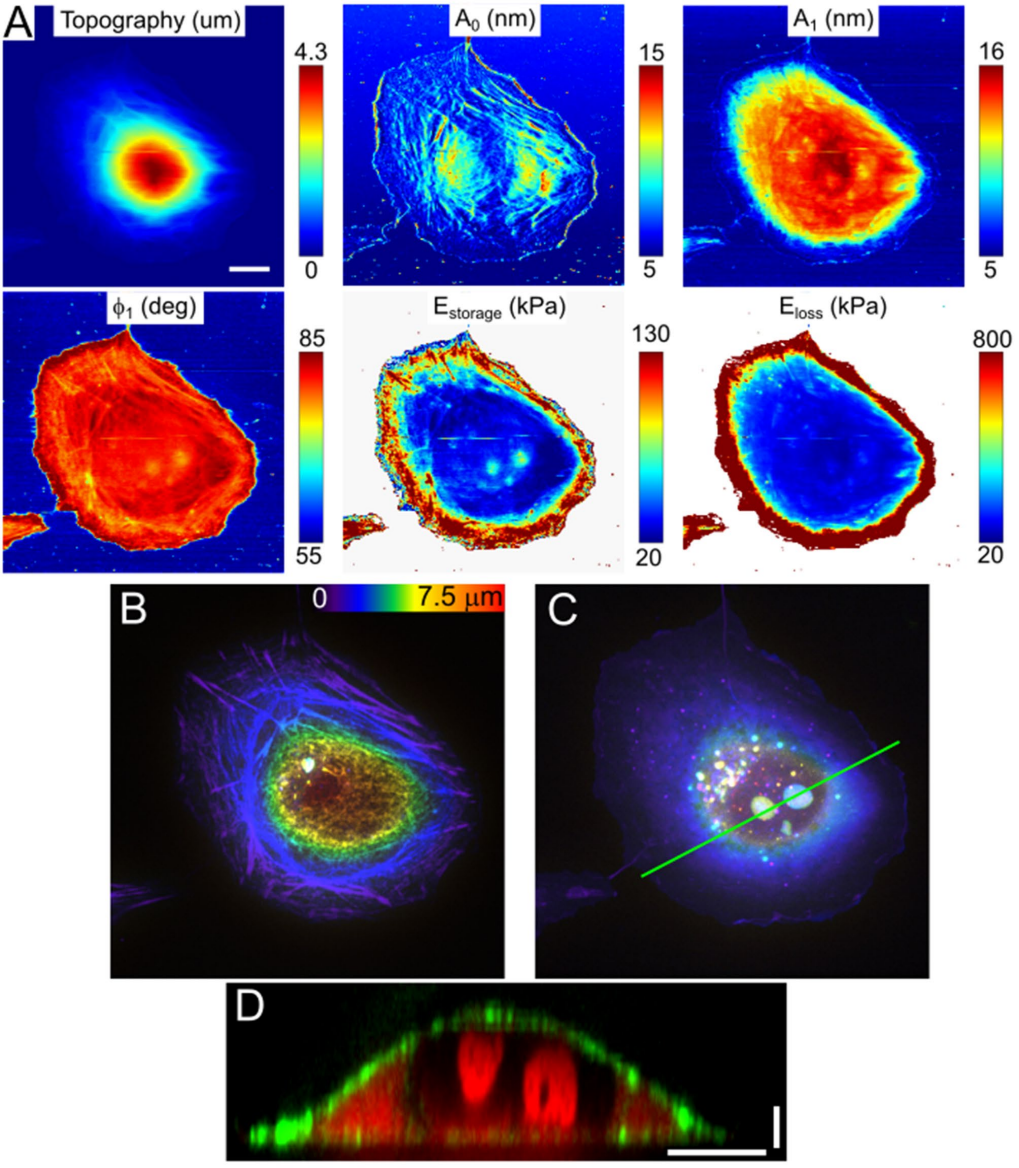
MDA-MB-231 breast cancer cell, AFM observables and nanomechanical maps (**A**) and SDCM color-coded height images (**B**,**C**). Some peripheral actin stress fibers and nucleoli could be detected in the amplitude and phase images, and in the E_storage_ map. Only the actin fibers, but not nucleoli, are presented in topography and deflection images. (**B**) Corresponding images of the actin cytoskeleton (SiR-actin staining) and nucleoli (SYTO85 staining), color-coded Z-stacks. (**D**) Vertical cross-section along the marked line shows the size and depth of the nucleoli (red) and cell shape (F-actin, green). Scale bars are 10 μm in the horizontal direction and 2 μm in the vertical direction.

**Figure 5:**
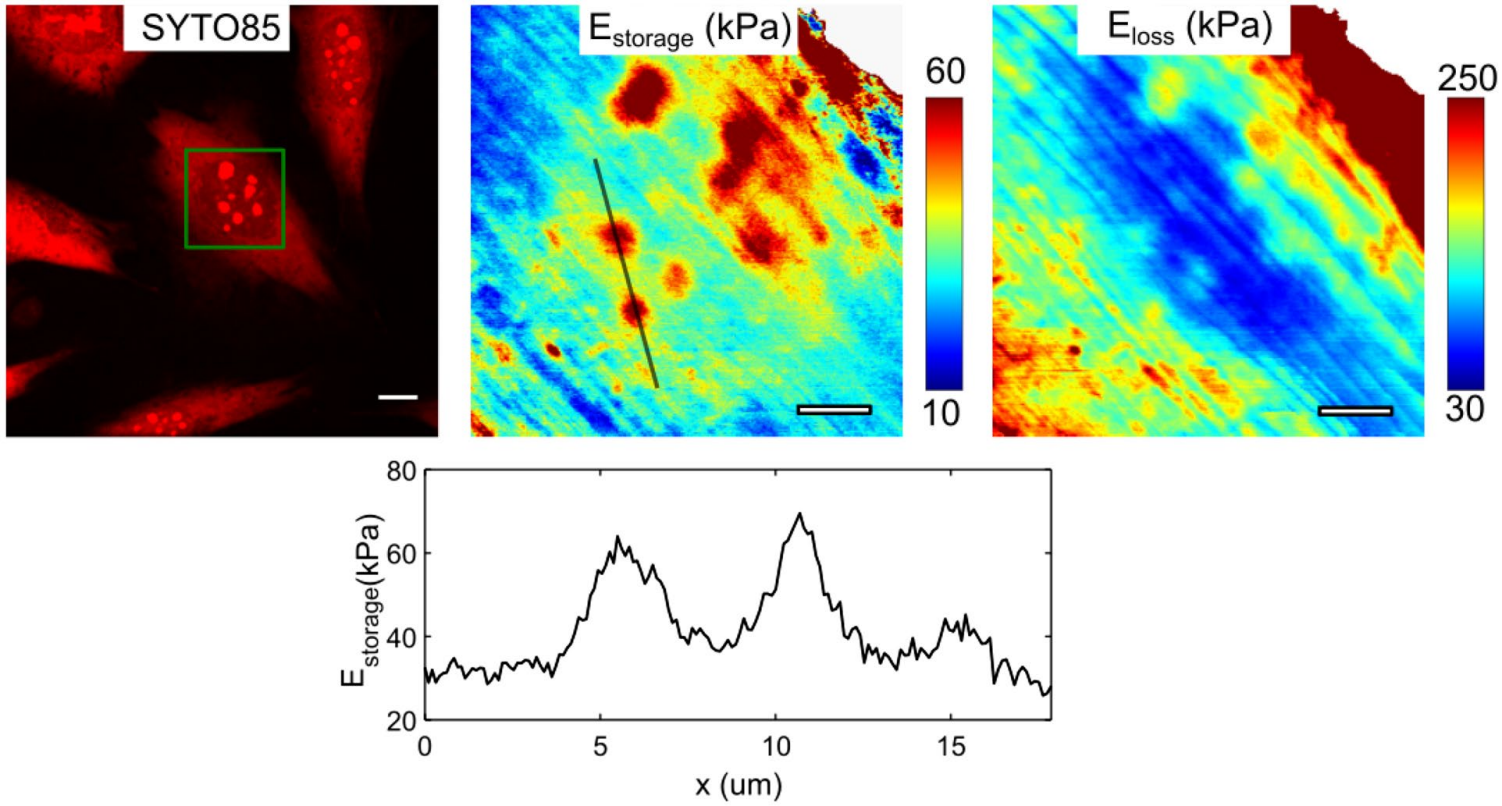
NIH 3T3 fibroblast, nanomechanical maps obtained over the nucleus region. Cross-section of the E_storage_ moduli over the two nucleoli (line on the map). E_storage_ above the nucleoli is about twice higher than above the surrounding area. Scale bars are 10 µm for the SDCM image, 5 µm for the nanomechanical maps.

The higher contrast achieved using the long tips allowed us to observe and identify different subcellular structures in the nanomechanical maps including stress fibers and nucleoli (Figs. 3, 4, 5) corresponding to a spatial resolution of ≈ 100–200 nm. Nucleoli are sites of ribosome biogenesis inside the nucleus. They could be easily detected in the phase and amplitude images, but not in the topography or deflection error images, thus confirming that the contrast is caused by mechanical properties of the buried object (Figs. 3A, 4A). We confirmed that these structures are indeed nucleoli by comparing AFM images with confocal fluorescence images of the same live cell stained for nucleic acids (Figs. 3B, 4B). Nucleoli can be detected as the brightest objects in such images. The nucleoli were seen previously with AFM subsurface imaging based on the analysis of deeper force curve segments^22,23^. The benefit of our setup is that the confocal z-stacks can be used to estimate the size and location of these organelles in the living cell. Vertical cross-sections over the nucleoli are presented in Fig. 3D with additional F-actin staining to visualize the cell borders. The depth of the nucleoli as measured from the z-stacks is up to several microns from the upper cell surface. Interestingly, the nucleus itself generally was not seen in the nanomechanical maps, indicating that it is not much stiffer than the surrounding cytoplasm.

Nucleoli were observed both in NIH 3T3 and MDA-MB-231 cells, in the latter cell type in smaller numbers but with a larger size (Figs. 3, 4). Detection of nucleoli from nanomechanical maps was hampered by the presence of stiff apical actin fibers in the NIH 3T3 cells (Fig. 5). From the nanomechanical maps, the effective storage Young’s modulus above the nucleoli was ~ 2 times higher than the modulus of the surrounding area for both types of cells. The primary function of the nucleolus is ribosome biogenesis, which is closely related to the metabolic state of a cell^10,53,54^. The nucleolus is a non-membranous cellular body and observed high stiffness could be caused by a high concentration of proteins and RNAs in a constrained space, which serves presumably to improve reaction and regulation efficiency. The size of the nucleolus positively correlates with ribosomal RNA synthesis, which in turn is governed by cell growth and metabolism. The larger size of nucleoli is characteristic of actively growing cancer cells^55^, which was observed here in MDA-MB-231 cells. Recent studies have also uncovered the role of the nucleolus in aging^53^ and establish the fact that nucleoli act as sensors of stress, regulating the cellular response to pathological insults^56^. While such studies mostly covered molecular alterations in the nucleoli, much less is known about their structural and mechanical properties. Some insights on structure-mechanics connection were obtained by studying isolated nucleoli^11^, but these findings remain to be confirmed in living cells.

Apical stress fibers were detected on nanomechanical maps of NIH 3T3 cells (Figs. 3, 5). They represent a special subtype of actin stress fibers called perinuclear actin cap and characterized by apical over-the-nucleus location with large adhesions at the basal cell surface (dome-like shape), connection with the nucleus via LINC complexes, and participation in mechanotransduction^57,58^. The perinuclear actin cap is presented in different cell types (mesenchymal stem cells, fibroblasts, endothelial cells) but is usually lost in cancerous cells. Due to its superficial apical location and high effective stiffness, the stress fibers from the perinuclear cap are often observed on the AFM images of cells taken in contact mode, both in topography and deflection error signal. They can also be distinguished in the phase images and on the nanomechanical maps (Fig. 3A).

### 3D nanomechanical characterization of sub-surface organelles

**Figure 6:**
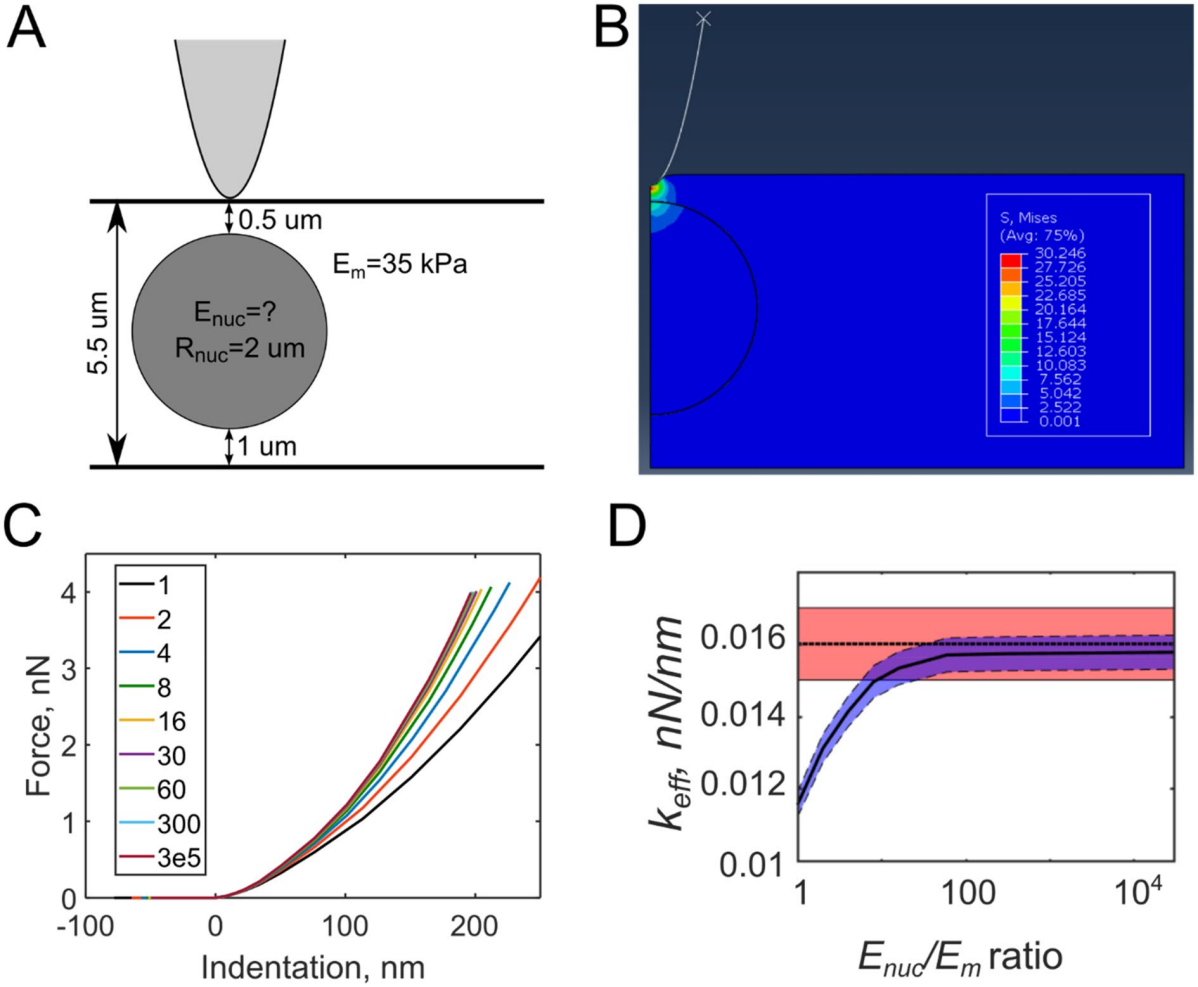
FEM analysis for estimating the mechanical properties of nucleoli. (**A**) Scheme of the FE model showing the dimensions extracted from the SDC images (the cantilever tip is not in scale). (**B**) Distribution of effective von Mises stress during the indentation over the nucleolus. (**C**) Simulated force-indentation curves, the legend shows *E*_*nuc*_/*E*_*m*_ ratio. The curves went steeper as the ratio increased. (**D**) The effective local stiffness *k*_*eff*_ (solid line) measured at the experimental value of force as a function of the *E*_*nuc*_/*E*_*m*_ ratio. It approaches the experimental *k*_*eff*_ value (dashed line, 0.016 ± 0.001 nN/nm, shaded area) at a high *E*_*nuc*_/*E*_*m*_ ratio (> 100). The 10% error (shaded area) is based on variation in *k*_*eff*_ value over nucleoli and surrounding nucleoplasm.

Next, we estimated the mechanical properties of nucleoli using the data available from both the AFM and SDCM experiments on the same cell (MDA-MB-231). A representative FE model was created based on confocal imaging, which accounted for the cell thickness, location and size of the selected nucleolus (Fig. 6A,B). An axisymmetric model with the indentation right above the nucleolus was created for simplicity, and only data for *E*_*storage*_ (storage modulus) and *k*_*eff*_ (local effective stiffness) were used. *k*_*eff*_ corresponds to the conservative force gradient (see supplementary materials for details) and, among other parameters, depends on the difference between the properties of nucleolus and the surrounding matrix. The goal was to estimate the modulus of the nucleolus based on the data from AFM nanomechanical maps (*F*_*ts*_, *E*_*storage,*_ and *k*_*eff*_ values) acquired directly above this nucleolus and in its vicinity. The value of the modulus of the matrix around the nucleoli was prescribed based on the values from the nanomechanical maps in the vicinity of the nucleoli (with bottom-effect correction and excluded data above the stress fibers). During the FE simulation, the modulus of the nucleoli *E*_*nuc*_ was continuously increased relative to the modulus of the surrounding nucleoplasm *E*_*m*_, and the force-indentation curves were recorded (Fig. 6C). Then, for each curve, the value of *k*_*eff*_ from the simulation was calculated at the experimental force *F*_*ts*_ (0.8 nN) and compared with the experimental value of *k*_*eff*_ (0.016 nN/nm) (Fig. 6D). From this analysis, we find that the simulated *k*_*eff*_ approaches the experimental one at high values of the nucleolus modulus (*E*_*nuc*_/*E*_*m*_ > 10). This means that nucleoli are much stiffer than the nucleoplasm with the lowest bound of an estimate of *E*_*storage*_ ≈ 100 kPa. The upper bound of the estimate, however, cannot be currently determined given the low stiffness of the selected cantilever (as selected for live-cell imaging) and its resulting inability to deform the nucleolus enough for quantitatively ascertaining the upper bound of the modulus measurement. Similar results were obtained for NIH 3T3 fibroblasts (data not shown). Therefore, we might conclude that the nucleolus is a very stiff organelle, probably due to high molecular packing density^11^. Nanomechanical estimations of buried objects in living cells might enable further studies in the area of cell physiology and toxicology.

## Conclusions

Streamlining AFM cantilever’s with long-carbon tips can significantly minimize their long-range hydrodynamic interactions with the cell surface, thus significantly enhancing the spatial resolution of nanomechanical maps and minimizing topography-related artifacts. Combining multi-harmonic AFM with simultaneous Spinning Disk Confocal Microscopy (SDC) and live-cell fluorescent markers enables the unambiguous correlation between observed heterogeneities in nanomechanical maps with specific subcellular structures. By integrating organelle size and depth data from SDC with heterogenous nanomechanical stiffness maps from multi-harmonic AFM and computational approaches, it is possible to quantitatively estimate the mechanical properties of sub-cellular structures such as nucleoli in living cells for the first time. This integration of dAFM, SDC, and computational approaches opens the door to mechanobiology of sub-cellular structures such as nucleoli, mitochondria, Golgi apparatus, and ribosomes, and relating changes in sub-cellular organelle properties with key cell metamorphosis or disease.

## Materials and methods

### Sample preparation

NIH 3T3 and MDA-MB-231 cell lines were maintained in Dulbecco’s modified Eagle’s medium (DMEM) containing 10% FBS and 1% antibiotic/antimycotic solution (Invitrogen, Carlsbad, CA) in a humidified 5% CO_2_ atmosphere at 37 °C. Prior to AFM experiments, cells were re-plated onto 50 mm glass-bottom cell culture dishes (FluoroDish, World Precision Instruments, Sarasota, FL) coated with 30 μg/mL fibronectin for 30 min (Sigma-Aldrich, St. Louis, MO) and grown for an additional period of 1–2 days to final confluency of ~ 60–70%. SiR-actin staining^59^ was performed according to the manufacturer’s protocol (Cytoskeleton, Inc., Denver, CO). Briefly, the cells were incubated with 200 nM SiR-dye and 10 µM verapamil (broad-spectrum efflux pump inhibitor improving the staining efficiency) diluted in regular growth media overnight. The cells were stained with CellMask Green plasma membrane staining solution (final concentration 5 ug/ml; Life Technology) and SYTO 85 Orange nucleic acid stain (5 µM; Life Technology) for 15 min right before AFM experiments, which were conducted after several washing steps with PBS in normal cell growth medium with 20 mM HEPES.

Polyacrylamide gel was prepared from stock solutions of acrylamide (40%, Sigma-Aldrich, USA) and bis-acrylamide (1%, Sigma-Aldrich, USA) in PBS and polymerized by addition of the photoinitiator Irgacure 2959 (0.5% w/v, (2-Hydroxy-4′-(2-hydroxyethoxy)-2-methylpropiophenone, Sigma-Aldrich, USA). Final concentrations of acrylamide (4%) and bis-acrylamide (0.2%, w/v) were chosen to prepare a PAAm gel with approximately 1–2 kPa Young’s modulus, which is close to the cell stiffness. Polymerization was done between two coverslips under 10 min of UV-exposure, prepared PAAm gels had a ~ 100 μm thickness. AFM measurements were performed in PBS containing 0.1% Triton X-100 detergent (Sigma-Aldrich, USA) to decrease probe-gel adhesion^60^. The Young’s modulus of the gel was calculated from AFM indentation experiments conducted with the CSC38 cantilevers (rectangular, Micromash Inc., Estonia) modified with 5 μm diameter silicon dioxide bead (Microspheres-Nanospheres, Corpuscular, NY, USA). Force curves were obtained at 4 µm/s piezo speed.

### Dynamic AFM material property mapping

We used the MFP-3D-Bio AFM (Asylum Research, an Oxford Instruments Company, Santa Barbara, CA) mounted on an IX-71 inverted optical microscope (Olympus, Tokyo, Japan) and integrated with an SDC microscope Andor Revolution XD (Andor Technology, South Windsor, CT). BL-TR400PB cantilevers (Olympus/Asylum Research) with a nominal spring constant of 0.09 N/m, a nominal tip radius of 42 nm (± 12 nm), and a half-opening angle α = 35° were used in our previous works. The modified version of these cantilevers with a 15-µm tall tip made of diamond-like carbon with 50–300 nm radius and 11° tilt compensation (nanotools USA LLC) was employed here. The value of cantilever spring constant was determined with laser Doppler vibrometry (Polytec MSA-400 Micro System Analyzer from Polytec GmbH, Waldbronn, Germany) from thermal vibrations in air using the equipartition theorem^61^. The cantilever was mounted on the iDrive Lorentz force excitation holder that enables direct excitation of the cantilever. Before and after measurements, the relationship between the photodiode signal and cantilever deflection (sensitivity factor S) was calibrated from the thermal tune spectra acquired far from the sample surface^62^. The AFM images were acquired with the feedback on the mean deflection to improve acquisition speed (1–2 Hz)^29^ with setpoint ~ 5–15 nm (force ~ 0.5–1.5 nN, corresponds to indentation of 50–200 nm). The acquisition time was 5–10 min per image. The amplitude far from the sample was selected to be approximately 10–20 nm. The conversion of AFM observables to the maps of mechanical properties was done according to^29^, the theory is reproduced briefly in Supplementary Material. The Hertz’s contact model for paraboloid indenter geometry was used; correction for the finite sample thickness was applied using the correction coefficients from^63^.

### Spinning disk confocal fluorescence imaging

The fluorescent images were acquired by a spinning-disk confocal microscope Andor Revolution XD with 100 × N.A. 1.4 oil immersion or 60 × N.A. water immersion objective on the Olympus IX-71 inverted optical microscope. Generally, the z-stacks were acquired before and after the acquisition of an AFM image of the same cell. The spacing between the optical sections was chosen based on the Nyquist criterion (0.21 μm for the used setup). The imaging parameters (laser intensity and exposition time) were adjusted in preliminary experiments to decrease the acquisition time and still preserve high signal-to-noise ratio and low phototoxicity. The fluorescent images were analyzed by Fiji (NIH, Bethesda, MD).

### Finite element simulations

Finite element (FE) simulations were conducted in Abaqus CAE (version 14, Simulia Corp., Providence, RI). The axisymmetric system was created based on the real dimension of the cantilever tip and the cell. The latter one was measured from SDC images. The material models used for both the nucleolus and surrounding matrix was a linear elastic material (isotropic, homogeneous, Poisson’s ratio = 0.49). The bottom surface of the sample was constrained; the contact between the indenter and the sample was considered to be frictionless. The mesh was refined in the contact region, and a mesh sensitivity study was conducted in advance.

## Supplementary Information

Supplementary Information.

Supplementary Information.

## Data Availability

The datasets generated during and/or analyzed during the current study are available from the corresponding author on reasonable request.
