# Supplementary material for: 3D nanomechanical mapping of subcellular and sub-nuclear structures of living cells by multi-harmonic AFM with long-tip microcantilevers
Source: Sci Rep. 2022 Jan 11;12:529. doi: 10.1038/s41598-021-04443-w (PMC8752865; doi:10.1038/s41598-021-04443-w)
Supplement: Supplementary file 1 — Supplementary Information. [file 41598_2021_4443_MOESM1_ESM.pdf]

Supplementary Information

3D nanomechanical mapping of subcellular and sub-nuclear structures of living cells by multi-harmonic AFM with long-tip microcantilevers

Yuri M. Efremov[1,2,3,4], Daniel M. Suter[2,5,6,7], Peter S. Timashev[3,4,8], Arvind Raman[1,2*]

[1]School of Mechanical Engineering, Purdue University, West Lafayette, Indiana, USA

[2]Birck Nanotechnology Center, Purdue University, West Lafayette, Indiana, USA

[3]Institute for Regenerative Medicine, Sechenov University, Moscow, Russia

[4]World-Class Research Center "Digital Biodesign and Personalized Healthcare," Moscow, Russia

[5]Department of Biological Sciences, Purdue University, West Lafayette, Indiana, USA

[6]Bindley Bioscience Center, Purdue University, West Lafayette, Indiana, USA

[7]Purdue Institute for Integrative Neuroscience, West Lafayette, Indiana, USA

[8]Chemistry Department, Lomonosov Moscow State University, Moscow, Russia

*Corresponding author. E-mail address: raman@purdue.edu

This supplementary documentation has been created to provide additional information to support the main text.

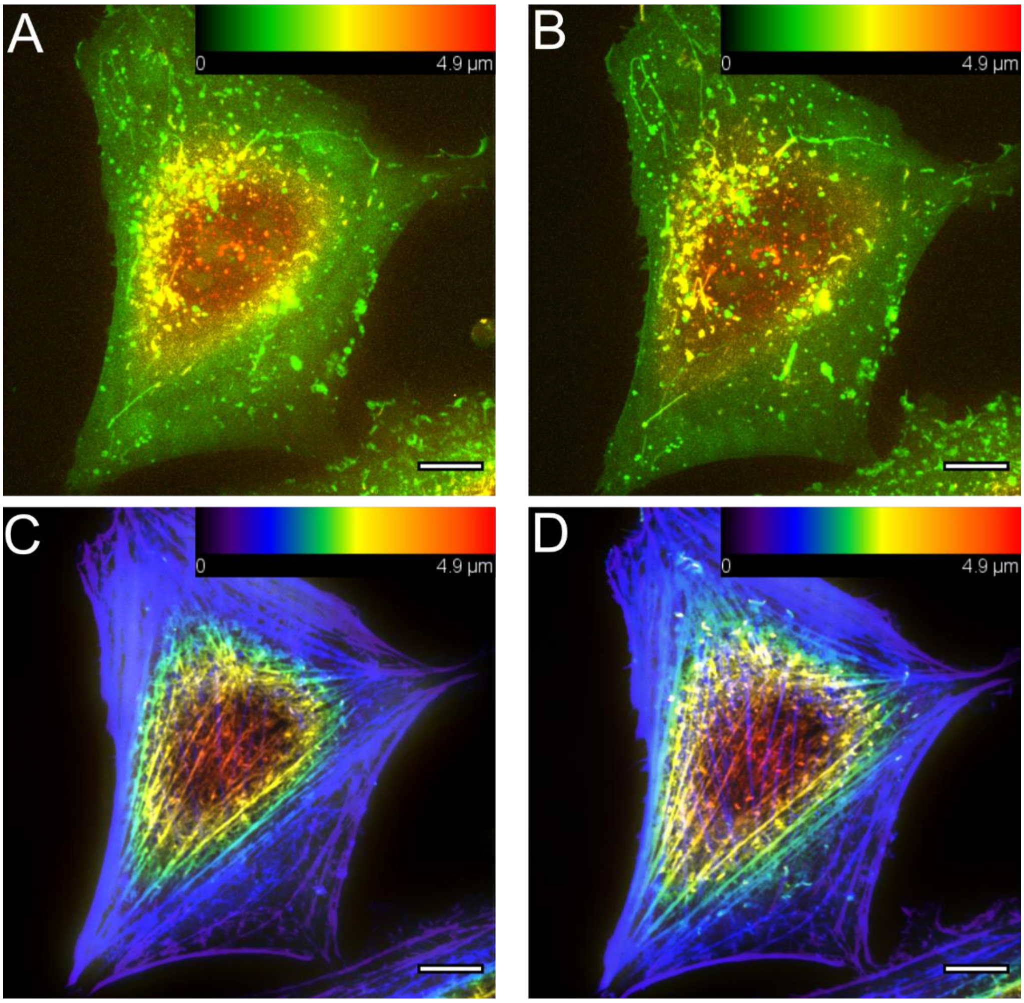

Figure S1. Images of the plasma membrane, CellMask Green staining (A, B), and actin cytoskeleton, SiR-actin staining (C, D), of NIH 3T3 fibroblast before (A, C) and after (B, D) scanning using a cantilever with a long carbon tip. Color-coded Z-stacks. No substantial changes were observed after the scanning.

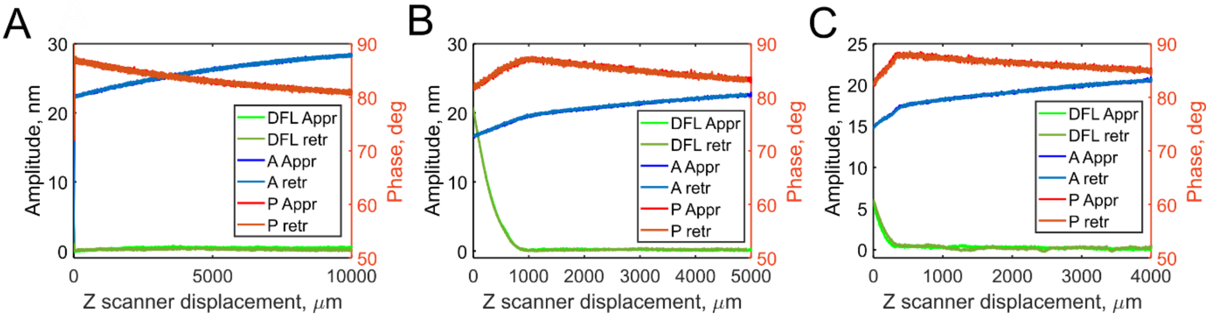

Figure S2. Examples of deflection (*DFL*), amplitude (*A*), and phase (*P*) versus Z scanner displacement curves acquired over glass, gel, and cell using a cantilever with a long carbon tip. Both approach (Appr) and retraction (Retr) curves are shown, which are very close to each other. (A) Curve acquired over the glass in the PBS. (B) Curve acquired over the hydrogel in the PBS. (C) Curve acquired over the NIH 3T3 fibroblast in the cell medium. There is no observable adhesion in the deflection retraction curve.

Theory of multi-harmonic imaging and spectroscopy

Here we briefly reproduce the theory for nanomechanical properties mapping developed in our previous works [28,29,31]. The theory accounts for (a) the difference in the resonant response of the cantilever when located far and near the sample surface due to viscous hydrodynamics, and (b) the dynamics of the harmonically oscillating cantilevers interacting with the sample surface. First consideration arises because the hydrodynamic loading changes both the natural frequency and the damping of the cantilever as it comes closer to the sample surface [31,41,64]. From linear vibration theory, the motion $q(t)$ of a point mass oscillator of natural frequency ω_n and quality factor Q_n excited by a harmonic force can be described as:

$$\frac{\ddot{q}}{\omega_n{}^2} + q + \frac{1}{\omega_n Q_n}\dot{q} = \frac{F_{mag}\, sin(\omega_{dr} t)}{k_{cant}}, \tag{S1}$$

where F_{mag} is the amplitude of the magnetic excitation force, ω_{dr} is the drive frequency, and k_{cant} is the equivalent stiffness of the first cantilever eigenmode. The steady state vibration response is:

$$q(t) = A\, sin(\,\omega_{dr} t - \phi),$$

$$A = \frac{F_{mag}}{k_{cant}} \frac{1}{\sqrt{\left(1 - \left(\frac{\omega_{dr}}{\omega_n}\right)^2\right)^2 + \left(\frac{\omega_{dr}}{Q_n \omega_n}\right)^2}},$$

$$tan\, \phi = \frac{\left(\frac{\omega_{dr}}{Q_n \omega_n}\right)}{\left(1 - \left(\frac{\omega_{dr}}{\omega_n}\right)^2\right)},$$

$$sin\, \phi = \frac{\left(\frac{\omega_{dr}}{Q_n \omega_n}\right)}{\sqrt{\left(1 - \left(\frac{\omega_{dr}}{\omega_n}\right)^2\right)^2 + \left(\frac{\omega_{dr}}{Q_n \omega_n}\right)^2}},$$

$$cos\, \phi = \frac{1 - \left(\frac{\omega_{dr}}{\omega_n}\right)^2}{\sqrt{\left(1 - \left(\frac{\omega_{dr}}{\omega_n}\right)^2\right)^2 + \left(\frac{\omega_{dr}}{Q_n \omega_n}\right)^2}}.. \tag{S2}$$

When the drive frequency is tuned to achieve maximum amplitude, the following relationships hold:

$$\frac{\omega_{dr}}{\omega_n} = \sqrt{1 - \frac{1}{2Q^2}},$$

$$A = \frac{F_{mag}}{k_{cant}} \frac{Q}{\sqrt{1 - \frac{1}{4Q^2}}},$$

$$tan\, \phi = \sqrt{4Q^2 - 2}. \tag{S3}$$

Far from the sample, the natural frequency and Q-factor of the cantilever are ω_{far} , Q_{far} . The drive excitation frequency is tuned to achieve maximum amplitude. Hence, Eq. S3 applies and we have the following relationships for the amplitude and phase far from the surface A_{1far} , ϕ_{1far} :

$$A_{1far} = \frac{1}{\sqrt{1-\frac{1}{4Q^2}}} \frac{F_{mag} Q_{far}}{k_{cant}},$$

$$\phi_{1far} = tan^{-1} \sqrt{4Q_{far}{}^2 - 2}. \tag{S4}$$

Note that when tuning the cantilever far from the sample, the phase lag at the frequency of peak amplitude is not to be set to $\pi/2$ or 90° rather it should be set to $\tan^{-1}\sqrt{4Q_{far}{}^2 - 2}$ which say for $Q = 2$ is a surprising 75°[30]. From Eq. (S4), the F_{mag} could be calculated as:

$$F_{mag} = \frac{k_{cant} A_{1far}}{Q_{far}} \sqrt{1-\frac{1}{4Q^2}}. \tag{S5}$$

When brought near the sample and prior to the tip-sample interaction, the natural frequency and Q-factor of the cantilever change to ω_{near}, Q_{near} and as a consequence the amplitude and phase also change to A_{1near}, ϕ_{1near} . Thus, the excitation frequency no longer corresponds to the drive frequency at which maximum amplitude occurs. Here, we invoke the more general Eqs. S2 and S3:

$$A_{1near} = \frac{F_{mag}}{k_{cant}} \frac{1}{\sqrt{(1-(r)^2)^2 + \left(\frac{r}{Q_{near}}\right)^2}},$$

$$sin\,\phi_{1near} = \frac{\left(\frac{r}{Q_{near}}\right)}{\sqrt{(1-r^2)^2 + \left(\frac{r}{Q_{near}}\right)^2}},$$

$$cos\,\phi_{1near} = \frac{1-r^2}{\sqrt{(1-r^2)^2 + \left(\frac{r}{Q_{near}}\right)^2}};$$

$$where,$$

$$r = \frac{\omega_{dr}}{\omega_{near}}. \tag{S6}$$

By rearranging above equations, we get:

$$1 - \left(\frac{\omega_{dr}}{\omega_{near}}\right)^2 = \frac{F_{mag}\, cos\,\phi_{1near}}{k_{cant} A_{1near}},$$

$$\frac{\omega_{dr}}{Q_{near}\omega_{near}} = \frac{F_{mag}\, sin\, \phi_{1near}}{k_{cant}A_{1near}};$$
$$or,$$
$$\omega_{near}{}^{2} = \frac{\omega_{dr}{}^{2}}{1 - \frac{F_{mag}\, cos\, \phi_{1near}}{k_{cant}A_{1near}}},$$
$$Q_{near} = \frac{F_{mag}\, sin\, \phi_{1near}}{k_{cant}A_{1near}} \sqrt{1 - \frac{F_{mag}\, cos\, \phi_{1near}}{k_{cant}A_{1near}}}. \quad \text{(S7)}$$

The equation of motion of the vibrating cantilever interacting with the sample becomes:

$$\frac{\ddot{q}}{\omega_{near}{}^{2}} + q + \frac{1}{\omega_{near}Q_{near}}\dot{q} = \frac{F_{mag}\, sin(\omega_{dr}t) + F_{ts}}{k_{cant}}, \quad \text{(S8)}$$

where F_{ts} is the tip-sample interaction force. Let the steady state motion of the tip interacting with the sample comprise of only the 0th and 1st and 2nd harmonics so that the tip displacement and velocity are:

$$q(t) = A_0 + A_1\, sin(\omega_{dr}t - \phi_1) + A_2\, sin(2\omega_{dr}t - \phi_2)$$
$$= A_0 + A_1\, sin(\theta) + A_2\, sin(2\theta + 2\phi_1 - \phi_2),$$

$$\dot{q}(t) = A_1\omega_{dr}\, cos(\theta) + 2A_2\omega_{dr}\, cos(2\theta + 2\phi_1 - \phi_2), \quad \text{(S9)}$$

where $\theta = \omega_{dr}t - \phi_1$. Depending on the properties of the sample, less number of the harmonics can govern the cantilever motion (e.g. just 0th and 1st), while higher harmonics are usually not detectable in the experiment. Here we consider this case specifically.

Since the tip motion is periodic, the tip-sample interaction force must be periodic. This leads to the following Fourier expansion of the tip-sample interaction force in terms of conservative and dissipative components:

$$F_{ts} = F_{ts,CONS}^{0} + \sum_{n=1}^{\infty} F_{ts,DISS}^{n}\, cos(n\theta) + \sum_{n=1}^{\infty} F_{ts,CONS}^{n}\, sin(n\theta), \quad \text{(S10)}$$

where $F_{ts,CONS}$ is the force conservative component (tip-sample position dependent), and $F_{ts,DISS}$ is the force dissipative component (tip velocity dependent). In the intermittent contact regime and while oscillating in permanent contact, it can be shown that, $F_{ts,CONS}(\theta)$ is symmetric about $\theta = 3\pi/2$ while $F_{ts,DISS}(\theta)$ is antisymmetric about $\theta = 3\pi/2$. As a result, while $F_{ts,CONS}^{1}(\theta)$ is the 1st Fourier *sine* coefficient, the $F_{ts,CONS}^{2}(\theta)$ on the other hand is the 2nd cosine coefficient since $\sin(\theta)$ and $\cos(2\theta)$ are both symmetric about $\theta = 3\pi/2$.

Substituting Eqs. (S10, S9) for the first two sine and cosine harmonic terms in the equation into (S8), using relations (S7) and balancing separately the constant, readily leads to the following results that link the Fourier components of the interaction forces to the observables (cantilever harmonics amplitudes and phases). The *n^th^* Fourier coefficient of the conservative interaction force is called the *n^th^* harmonic virial and the *n^th^* Fourier coefficient of the dissipative interaction force is called the *n^th^* harmonic dissipation:

$$F^0_{ts,CONS} = k_{cant}A_0,$$

$$F^1_{ts,CONS} = \frac{k_{cant}A_{1far}}{Q_{far}}\left(-cos(\phi_1) + \frac{A_1}{A_{1near}}cos(\phi_{1near})\right)\sqrt{1-\frac{1}{4Q^2_{far}}},$$

$$F^1_{ts,DISS} = \frac{k_{cant}A_{1far}}{Q_{far}}\left(-sin(\phi_1) + \frac{A_1}{A_{1near}}sin(\phi_{1near})\right)\sqrt{1-\frac{1}{4Q^2_{far}}}, \quad \text{(S11)}$$

Let us annotate the dynamic tip indentation into the sample as

$$\delta(t) = -(Z+q), \quad \text{(S12)}$$

and the average tip indentation as

$$\delta_0 = -Z - A_0. \quad \text{(S13)}$$

Next, in recognition of the experimental observation that the tip oscillation is much smaller compared to the net average indentation δ_0 on a sample we describe the interaction forces as a Taylor series in $\delta - \delta_0$ and neglect the contribution of the higher order terms:

$$F_{ts} = F_{ts}(\delta_0) + k^{dynamic}_{sample}(\delta - \delta_0) + c^{dynamic}_{sample}\dot{\delta}, \quad \text{(S14)}$$

where $k^{dynamic}_{sample}$ (N/m) and $c^{dynamic}_{sample}$ (N-s/m) respectively are the conservative force gradient
(stiffness) and damping at that particular indentation value. All these parameters typically depend on the applied mean force and drive frequency.
From Esq. S14 and S10, the Fourier coefficients of the interaction force could be evaluated in terms of the local properties:

$$F^0_{ts,CONS} = F_{ts}(\delta_0),$$
$$F^1_{ts,CONS} = -k^{dynamic}_{sample}A_1,$$
$$F^1_{ts,DISS} = -c^{dynamic}_{sample}\omega_{dr}A_1, \quad \text{(S15)}$$

Thus, the observables $F^1_{ts,CONS}$ and $F^1_{ts,DISS}$ can be used to determine the effective sample stiffness $k^{dynamic}_{sample}$ and damping $c^{dynamic}_{sample}$ at a mean indentation δ_0, while the observable $F^0_{ts,CONS}$ measures the force needed to maintain a constant amplitude reduction due to local stiffness and damping. The fact that at each point on the image we know the mean force applied and can extract the effective sample stiffness

and damping allows us to estimate quantitatively the local mechanical properties such as the local elastic modulus.
For soft objects like living cells the second and higher harmonic amplitudes are usually undetectable. Therefore, the interaction force can be described as:

$$F_{ts} = F_{ts}(\delta_0) - k_{sample}^{dynamic} A_1 \, sin(\theta) - c_{sample}^{dynamic} A_1 \omega_{\,dr} cos(\theta). \qquad \text{(S16)}$$

We can use equations S15 and S11 to calculate $F_{ts}(\delta_0)$, $k_{sample}^{dynamic}$, and $c_{sample}^{dynamic}$ from the experimental observables:

$$F_{ts,CONS}^{0} = k_{cant} A_0,$$

$$k_{sample}^{dynamic} = \left(\frac{k_{cant} A_{1far}}{Q_{far} A_1} cos\,\phi_1 - \frac{k_{cant} A_{1far}}{Q_{far} A_{1near}} cos\,\phi_{1near} \right) \sqrt{1 - \frac{1}{4Q_{far}^2}},$$

$$c_{sample}^{dynamic} = \left(\frac{k_{cant} A_{1far}}{Q_{far} A_1 \omega_{dr}} sin\,\phi_1 - \frac{k_{cant} A_{1far}}{Q_{far} A_{1near} \omega_{dr}} sin\,\phi_{1near} \right) \sqrt{1 - \frac{1}{4Q_{far}^2}}, \qquad \text{(S17)}$$

These equations apply for both AM-AFM observables and also for deflection modulation (contact mode with resonant excitation) observables.

Now, we present in further detail the method to quantify the local mechanical properties by combining the experimental multi-harmonic observables 0th and 1st data on live cells. Local force and damping gradients allows the extraction of unknown constitutive material properties, which are more fundamental physical properties of cells, by using a tip-sample contact mechanics model of interest [29,31]. The Hertz's contact mechanics model with a linear viscoelastic expansion was used here [37]. The viscoelastic Hertz's model for a paraboloid-shaped AFM tip is:

$$F_{ts} = \frac{4}{3} \sqrt{R} E^* \delta_0^{\frac{3}{2}}, \qquad \text{(S18)}$$

with $E^* = \frac{E_{Hertz}^{storage}(\omega_{dr})}{(1-\nu^2)} + i \frac{E_{Hertz}^{loss}(\omega_{dr})}{(1-\nu^2)}$, the complex effective sample modulus consisting of an elastic storage $E_{Hertz}^{storage}$, and viscous loss E_{Hertz}^{loss} modulus representing the linear viscoelasticity of the sample evaluated at an average indentation depth (Pa); R, radius of the probe; ν, the Poisson's ratio of the sample; and δ_0, average indentation [65].

Using Eq. S11 for small oscillations assumptions as above and neglecting the contribution of the higher order terms in F_{ts} Taylor series expansion and in the multiplicative correction, we find that:

$$k_{sample}^{dynamic} = 2\sqrt{R\delta_0} \frac{E_{Hertz}^{storage}(\omega_{dr})}{(1-\nu^2)},$$

$$c_{sample}^{dynamic} \omega_{dr} = 2\sqrt{R\delta_0} \frac{E_{Hertz}^{loss}(\omega_{dr})}{(1-\nu^2)}. \qquad \text{(S19)}$$

Therefore, the $E_{Hertz}^{storage}$ and E_{Hertz}^{loss} values are related to $k_{sample}^{dynamic}$ and $c_{sample}^{dynamic}$ which were found above. However, value of the mean indentation δ_0 is required for the calculation. From Eqs. S15, S18 and S19 we can find:

$$\delta_0 = \left(\frac{3}{4}\frac{(1-\nu^2)}{\sqrt{R}\,E_{Hertz}^{storage}}F_{ts,CONS}^{0}\right)^{\frac{2}{3}},$$

$$E_{Hertz}^{storage} = \left(\left(\frac{1}{6}\right)^{\frac{1}{3}}\frac{F_{ts,CONS}^{1}}{A_1\left(F_{ts,CONS}^{0}\right)^{\frac{1}{3}}}\right)^{\frac{3}{2}}\frac{(1-\nu^2)}{\sqrt{R}},$$

$$E_{Hertz}^{loss} = \left(\left(\frac{1}{6}\right)^{\frac{1}{3}}\frac{F_{ts,DISS}^{1}}{A_1\left(F_{ts,CONS}^{0}\right)^{\frac{1}{3}}}\right)^{\frac{3}{2}}\frac{(1-\nu^2)}{\sqrt{R}}. \qquad \text{(S20)}$$

Hertz's contact mechanics model requires small sample indentations <10% of sample height. However, a model that takes into account the artifact generated by moderate and large indentations of paraboloidal tips in AFM measurements on thin samples and adherent cells is required. In this case, we chose to use the BECC contact model [39], which is a multiplicative analytical correction done to the commonly used Hertz's model. This model that takes into consideration topographical effects by large indentations in soft samples like living cells. We use here again a linear viscoelastic model to extract the constitutive material properties (oscillation amplitude is small compared to indentation). Thus, the resulting tip-sample interaction force model is:

$$F_{ts} = \frac{4}{3}\sqrt{R}E^*\delta_0^{\frac{3}{2}}\left(1 + a_1\left(\frac{\sqrt{R\delta}}{h}\right) + a_2\left(\frac{\sqrt{R\delta}}{h}\right)^2 + O\left(\frac{\sqrt{R\delta}}{h}\right)^3\right) \qquad \text{(S21)}$$

where the complex effective sample modulus E^* consisting of an elastic storage $E_{BECC}^{storage}$ and viscous loss E_{BECC}^{loss} modulus representing the linear viscoelasticity of the sample evaluated at an average indentation depth. δ, h, and R, respectively, are the indentation, the height of the sample at that location, and the probe radius. a_1 and a_2 are coefficients from the multiplicative analytical correction [39]. Note that the sample height could be calculated from the topography data as

$$h = Z + \delta_0. \qquad \text{(S22)}$$

Using Eq. S16 for small oscillations assumptions as previously presented and neglecting the contribution of the higher order terms in F_{ts} Taylor series expansion and in the multiplicative correction, we find that:

$$k_{sample}^{dynamic} = \frac{4}{3}\sqrt{R\delta_0}\frac{E_{BECC}^{storage}(\omega_{dr})}{(1-\nu^2)}\left(\frac{3}{2} + 2a_1\left(\frac{\sqrt{R\delta_0}}{h}\right) + \frac{5}{2}a_2\left(\frac{\sqrt{\delta R_0}}{h}\right)^2\right),$$

$$c_{sample}^{dynamic}\omega_{dr} = \frac{4}{3}\sqrt{R\delta_0}\frac{E_{BECC}^{loss}(\omega_{dr})}{(1-\nu^2)}\left(\frac{3}{2} + 2a_1\left(\frac{\sqrt{\delta R_0}}{h}\right) + \frac{5}{2}a_2\left(\frac{\sqrt{\delta R_0}}{h}\right)^2\right). \qquad \text{(S23)}$$

From S15, S21-S23 we can find:

$$F_{ts,CONS}^{0} = \frac{4}{3}\sqrt{R}\frac{E_{BECC}^{storage}}{(1-\nu^2)}\delta_0^{\frac{3}{2}}\left(1 + a_1\left(\frac{\sqrt{R\delta_0}}{h}\right) + a_2\left(\frac{\sqrt{R\delta_0}}{h}\right)^2\right),$$

$$F_{ts,CONS}^{1} = -\frac{4}{3}\sqrt{R}\frac{E_{BECC}^{storage}}{(1-\nu^2)}\delta_0^{\frac{1}{2}}\left(\frac{3}{2} + 2a_1\left(\frac{\sqrt{R\delta_0}}{h}\right) + \frac{5}{2}a_2\left(\frac{\sqrt{\delta R_0}}{h}\right)^2\right)A_1,$$

$$F_{ts,DISS}^{1} = -\frac{4}{3}\sqrt{R}\frac{E_{BECC}^{loss}}{(1-\nu^2)}\delta_0^{\frac{1}{2}}\left(\frac{3}{2} + 2a_1\left(\frac{\sqrt{R\delta_0}}{h}\right) + \frac{5}{2}a_2\left(\frac{\sqrt{R\delta_0}}{h}\right)^2\right)A_1. \qquad \text{(S24)}$$

These expressions link the experimental observables to the effective nanoscale mechanical parameters $E_{BECC}^{storage}$ and E_{BECC}^{loss}. A MATLAB code has been written that performs a nonlinear least squares best fit of those unknown nanomechanical properties δ_0, $E_{BECC}^{storage}$ and E_{BECC}^{loss} that best match the measured force harmonics [28,29]. It is important to keep in mind that these equations actually extract the effective properties of the live cell at a specific mean indentation δ_0 and excitation frequency ω_{dr}. With the above briefly discussed theory and the maps of multi-harmonic amplitudes and phases (A_0, A_1, and ϕ_1) it's possible to map the mean indentation (δ_0) and the complex elastic modulus of the viscoelastic sample ($E_{BECC}^{storage}$, and E_{BECC}^{loss}).
