# Supplementary material for: 3D nanomechanical mapping of subcellular and sub-nuclear structures of living cells by multi-harmonic AFM with long-tip microcantilevers
Source: Sci Rep. 2022 Jan 11;12:529. doi: 10.1038/s41598-021-04443-w (PMC8752865; doi:10.1038/s41598-021-04443-w)
Supplement: Supplementary file 2 — Supplementary Information. [file 41598_2021_4443_MOESM2_ESM.docx]

**Supplementary references**

64. Xu, X., Carrasco, C., de Pablo, P. J., Gomez-Herrero, J. & Raman, A. Unmasking imaging forces on soft biological samples in liquids when using dynamic atomic force microscopy: a case study on viral capsids. Biophys. J. 95, 2520–8 (2008).

65. Alcaraz, J. et al. Microrheology of human lung epithelial cells measured by atomic force microscopy. Biophys. J. 84, 2071–2079 (2003).
